# Evaluation of a Mobile App to Assist Patient Education and Research in Arthroplasty

**DOI:** 10.1016/j.artd.2024.101549

**Published:** 2024-11-16

**Authors:** Leina Suzuki, Francis Connon, Selin Munir, Sarah Piplica, Hemant Pandit, Daevyd Rodda

**Affiliations:** a Medacta Australia Pty Ltd, Lane Cove, New South Wales, Australia; b Department of Orthopaedic Surgery, Sunshine Coast University Hospital, Birtinya, Queensland, Australia; c School of Health, University of Sunshine Coast, Birtinya, Queensland, Australia; d Fortius Institute for Musculoskeletal Research, Birtinya, Queensland, Australia; e UniSC Clinical Trials, University of Sunshine Coast, Sippy Downs, Queensland, Australia; f Leeds Institute of Rheumatic and Musculoskeletal Medicine, University of Leeds, Leeds, UK

**Keywords:** ePROMs, Smartphone, App, Postoperative care, Patient education

## Abstract

**Background:**

Paper-based patient-reported outcome measures (PROMs) and patient education can assist in improving outcomes but is administratively burdensome. Mobile phone applications (‘apps’) can distribute extensive information and PROMs at relevant time points. This study aimed to assess the suitability of an app to guide postoperative management and record PROMs based on satisfaction and compliance.

**Methods:**

Thirty-four patients who were scheduled for a total hip/knee arthroplasty were enrolled into the study. Automatic notifications were sent by the app to complete PROMs at the appropriate time points. Patients were reminded via phone call if PROMs were not completed. An app satisfaction questionnaire was also completed, where a high score represented satisfaction with the app.

**Results:**

Patients remained satisfied with the app throughout the study with a mean score of 19.0 out of 25. 57% found the app to be helpful with completing surveys, with 63% preferring the app over paper handouts. Majority of the participants (68%) stated that they would use the app again. There was an overall mean compliance of 78% at all time points. Most patients (82%) required at least one phone call reminder, with 18% of patients completing their PROMs prompted by the app notification alone.

**Conclusions:**

A mobile phone app can be useful for both distributing patient education and collecting PROMs. PROMs collected using a mobile phone app still caused some administrative burden with many participants requiring multiple reminders to complete their questionnaires. While paper-forms will still be required for some patients, most found the app preferable to paper-form.

## Introduction

Patient engagement is the process of encouraging patients to be self-motivated and educated in their own care [1]. Patient education can help decrease stress and anxiety associated with surgery and thereby assist patients to manage their expectations. As such, studies have proposed patient education and thus engagement could improve outcomes and overall satisfaction postsurgery [[2], [3], [4]]. However, distributing educational materials in paper forms can be impractical as it lacks engagement, can be easily misplaced, as well as being administratively burdensome.

The growth of Mobile Health (mHealth) technology has been seen across the medical industry, designed to encourage patients to be educated and autonomous in their own health before and after surgery by systematically distributing educational materials. Models such as short message service bot and automated emails can be an effective method in delivering appropriate information and exercises in a timely manner. Other mHealth technologies have built in the ability for patients to have direct access and communication to the medical team, thereby allowing the medical team to collect information on the patient’s recovery and identify any unexpected complications.

Patient-reported outcome measures (PROMs) play a crucial role in assessing the effectiveness of clinical intervention by providing clinicians insight into the patient’s perception of their progress at various time points following surgery [5]. Traditionally, PROMs have been captured using paper-based questionnaires during clinician visitation. This approach to data collection requires a large reliance on administrative management to maintain collection compliance. Risks associated with paper data collection include incomplete or partial completion, illegibility, and invalid data entry. Electronic patient-reported outcome measures (ePROMs) can help mitigate the risks associated with paper-based questionnaires, by providing a platform for the patients to complete the questionnaires in their own time and with their own device. Whilst paper-based PROMs can be resource intensive requiring the clinicians or their clinic staff to track time points, disseminate PROMs, and follow-up patients, ePROMs can employ automated scheduled prompts to remind patients to complete the PROMs at the appropriate time, thereby reducing the risk of incomplete PROMs. Consistency and accuracy of data entry can be improved with ePROMs by allowing patients to enter data directly into the system. This can prevent the risk of incorrect data entry and reduce the cost of requiring research personal, making ePROMs a cost-effective option. ePROMs also allow patients to access their questionnaires remotely thereby aiding clinicians to capture a larger cohort, regardless of their geographic location.

This study aims to assess the suitability of a mobile phone application (app) to deliver postoperative education and record PROMs data by determining the overall patient satisfaction of using an app. The secondary aim is to determine the patient compliance of PROMs when ePROMs are embedded into an app.

## Material and methods

Human Research Ethics Committee approval was obtained prior to the commencement of the study. Between July 2020 and March 2021, patients scheduled for a total hip arthroplasty (THA) or total knee arthroplasty (TKA) were considered in the study. In this observational, prospective case series, all patients were recruited at a single site by the operating orthopedic surgeons (F.C., D.R.). Patients were included in the study if they had a compatible smartphone and were considered capable of using the mobile phone application by either of the 2 investigators by asking questions regarding their use of other apps on their smartphones. Once the patient had provided written informed consent, they were shown in person how to download and use the mobile phone based application (Patient Optimized Pathway app ‘POP-App,’ Medacta International SA, Switzerland). The app is available for both android and iPhone Operating System mobile devices. The patients were then asked to complete their preoperative PROMs using the application, with a reminder set at 30 days prior to the date of surgery.

### Postoperative

All patients were contacted the day after their surgery to ensure that they were not experiencing any difficulties in using the application. Postoperative treatment regime and exercises were provided to the patient through the app after the date of surgery. This included 6-7 postoperative exercises specific to THA/TKA. A notification was sent by the app at 2 weeks, 6 weeks, 3 months, and 6 months postoperatively to remind patients to complete their questionnaires. Patients were reminded via a phone call if PROMs were not completed at the required time point. At the final 6 months’ time point, patients were contacted and were welcomed to delete or keep the app.

### Questionnaires

All patients were required to complete PROMs using the app. Patients receiving a THA were asked to complete the Hip disability and Osteoarthritis Outcome Score and the EuroQol-5 dimension at all time points. The Oxford Hip Score was asked to be completed at all, except the final 6 months’ time point. Patients receiving a TKA were asked to complete the Knee injury and Osteoarthritis Outcome Score and the EuroQol-5 dimension at all time points. The Oxford Knee Score was asked to be completed at all, except the final 6 months’ time point. The compliance rate was calculated by recording the number of PROMs collected at each time point.

All patients were also asked to complete a satisfaction survey containing questions about the mobile app at all time points. The survey included 8 questions (Appendix 1) including 5 questions assessing patient satisfaction with the App and 3 asking their input on further improvements and willingness to use the App in future. The questions assessed the patient satisfaction toward the helpfulness of the app in providing rehabilitation material, access to educational material, ease of PROMs completion access to activity levels, as well as overall satisfaction with the app. The replies were measured on a Likert scale ranging from 1 (least amount of satisfaction) and 5 (highest level of satisfaction). The scores for each category were summed for a total satisfaction score, with the highest possible score being 25. Patients with a mean score of greater than or equal to 15 were considered to be satisfied with the app whereas patients with a score of less than 15 were considered to be dis-satisfied with the app. Patients with scores greater than or equal to 20 were considered highly satisfied.

Data was analyzed using Microsoft Excel (Microsoft Corporation, Redmond, WA). As this was a pilot study with a small sample size, no statistical significance was set. The mean, minimum and maximum scores of the PROMs and patient satisfaction questionnaires were reported for each time point as well as overall.

## Results

Forty-one patients were enrolled into the study, with 7 patients excluded from the study. Of the 7 withdrawn participants, 5 were withdrawn as they did not meet the study eligibility criteria, and 2 withdrew consent. A final study cohort of 34 patients were included for analysis. The average age of the cohort was 67.0 ± 8.7 years and 48% of the participants were male.

### Mobile app satisfaction

The mean scores at each time point were 20.4 (13-25) preoperatively, 18.7 (12-25) at 2 weeks, 17.8 (12-25) at 6 weeks, 19.2 (10-25) at 3 months, and 18.8 (12-25) at 6 months. The mobile app satisfaction score demonstrated consistent satisfaction at all time points, with the mean satisfaction score of 19.0 out of 25 for the entire duration of the study; 87% (83%-90%) of patients were considered satisfied, using the threshold of a score greater than or equal to 15, throughout the duration of the study; 50% (38%-61%) of patients were considered highly satisfied at all time points, with a score of greater than or equal to 20.

**Figure 1:**
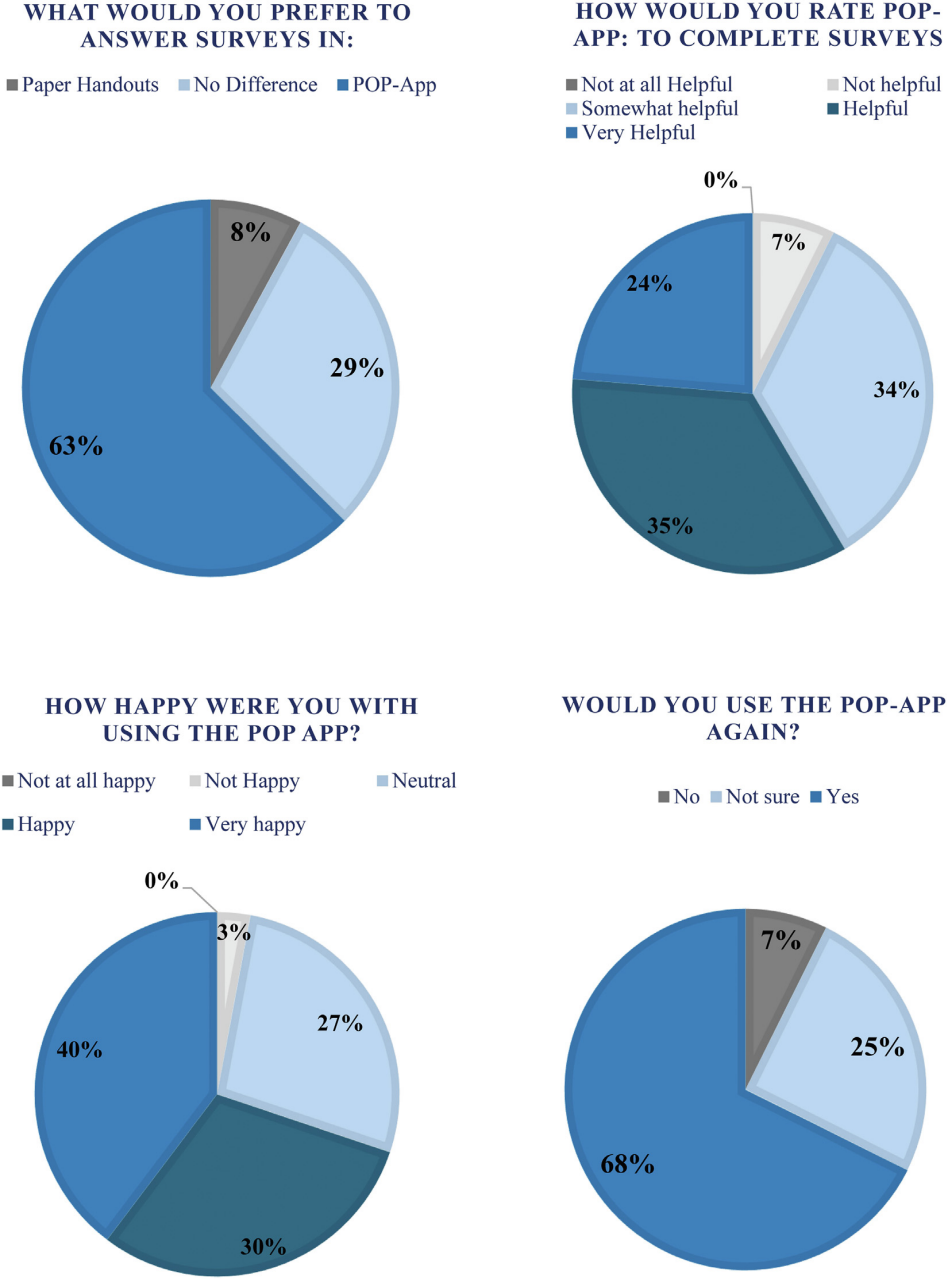
Graph representing patient satisfaction survey results where each pie graph represents a question from the survey. POP-App, Patient Optimized Pathway Application.

The results of the patient satisfaction questionnaire across all time points are displayed in Figure 1. Overall, 57% of participants reported the app to be either helpful or very helpful when completing surveys; 70% of participants were either happy or very happy with using the mobile app; 63% of participants preferred the app over paper handouts. The educational material and rehab requirements provided by the app were considered either helpful or very helpful by majority of the participants (59% and 56%, respectively); 68% of the participants stated that they would use the app, if they were to have this surgery again.

### PROMs compliance

**Figure 2:**
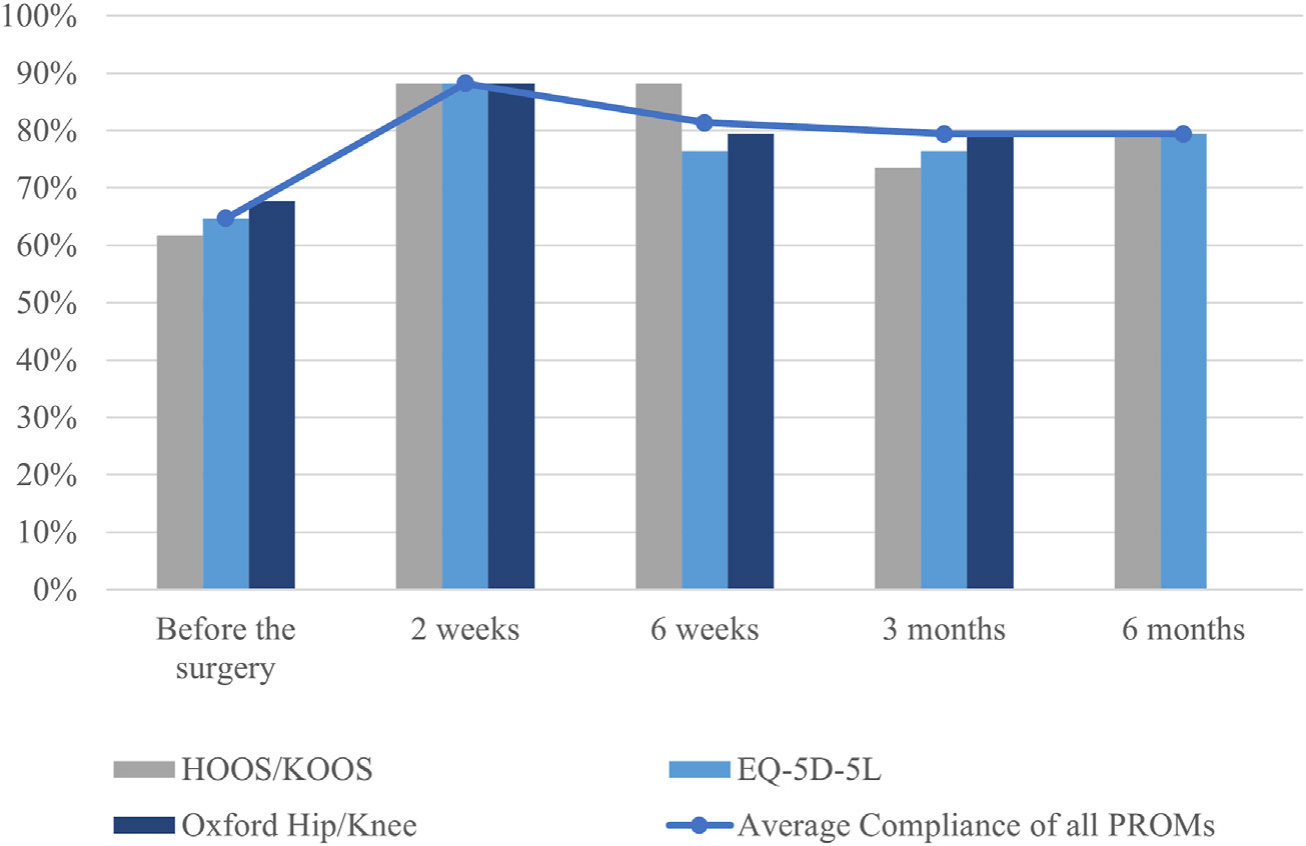
PROMs compliance at each time point. HOOS, Hip disability and Osteoarthritis Outcome Score; KOOS, Knee injury and Osteoarthritis Score. HOOS, Hip disability and Osteoarthritis Outcome Score; KOOS, Knee Injury and Osteoarthritis Outcome Score; PROMs, patient-reported outcome measures.

The overall PROMs compliance from preoperative to postoperative time point was 78% (65%-88%). The mean compliance for preoperative PROMs was 65%. The postoperative compliance for each time point was 88% at 2 weeks, 81% at 6 weeks, 79% at 3 months, and 79% at 6 months. The compliance of each PROMs questionnaire is shown in Figure 2.

**Figure 3:**
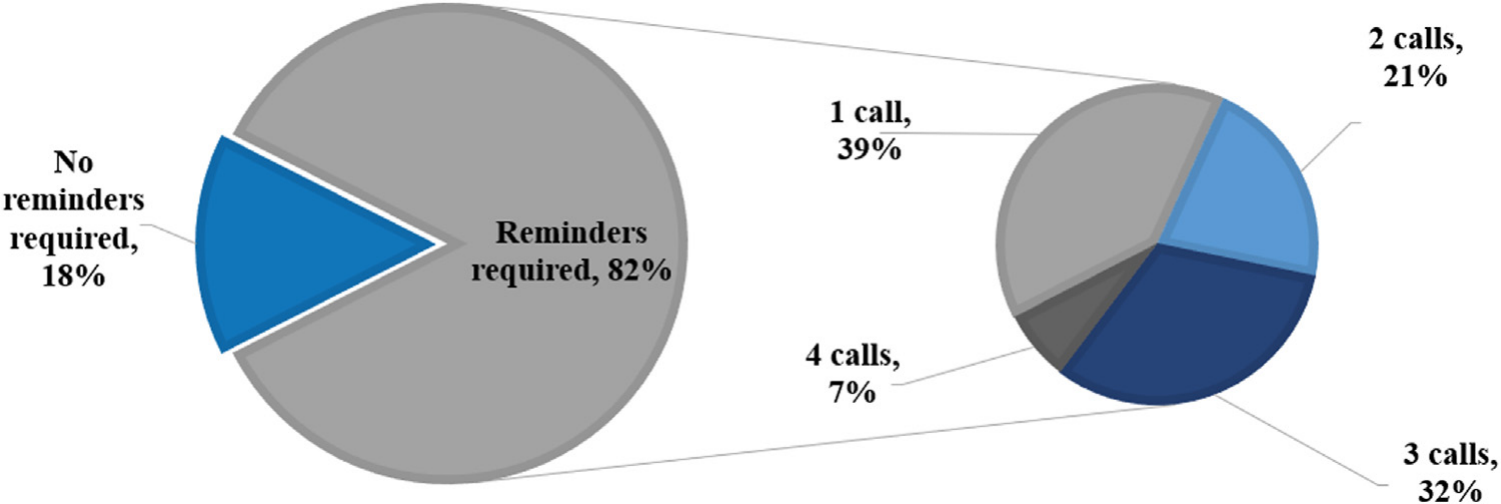
Graph representing percentage of follow-up calls required.

Most patients (82%) required at least 1 phone call reminder, with only 18% of patients completing their PROMs prompted by the app notification alone, as shown in Figure 3. There was no difference in mean age in patients that completed PROMs without a phone call reminder compared with patients that did require a phone call reminder with a mean age of 67.0 years and 68.5 years, respectively. Of the patients that required a phone call reminder, over the life of the study 39% required 1 phone call, 21% required 2 calls, 32% required 3 calls, and 7% required 4 calls. The average success rate of PROMs being completed after a phone call was 83% at 2 weeks, 40% at 6 weeks, and 69% at 3 months.

## Discussion

The use of mHealth technology has been adopted in a number of medical fields for its utility as a postoperative management tool. The main benefit of utilizing mHealth technologies is their ability to provide an easy distribution of information in a timely manner and enable patients the ability to conduct their postoperative treatment remotely.

Studies have found mHealth can help patients raise symptoms promptly to the medical team, which can assist in the detection of expected and unexpected side effects [[6], [7], [8]]. With the increasing demand for alternative health services in the orthopedic industry, there has been a growth in the use of mHealth to facilitate remote monitoring without compromising the quality of health care provided. Remote monitoring can be in the form of tracking exercises through step counts, where early mobilization plays a crucial role in postoperative recovery, to self-directed physical therapy. In a randomized controlled trial, Tripuraneni et al. [9] found no difference when comparing the postoperative PROMs and clinical outcomes of patients with self-directed physical therapy through mHealth technology and patients with traditional in-clinic physical therapy. Studies have found that cohorts with access to some form of remote monitoring through mHealth had less rehospitalization [10] or unplanned visitations [11] compared to standard care. In this study, there was a relatively even split with participants’ satisfaction toward the rehabilitation information provided through the app, with 56% finding the information to be useful. This is likely due to the patients also receiving standard physical therapy provided as part of the treating surgeon’s standard of care. As such, the generic rehabilitation exercises provided by the app may not have added a significant amount of value to the in-person rehabilitations that the patient were already receiving.

PROMs are used by clinicians to investigate the effects of a surgical intervention and compare outcomes between populations on a wider scale. PROMs can also act as a form of patient monitoring, as the results from the PROMs questionnaire can be used to provide patient-centered care where the clinician may choose to modify their treatment based on the PROMs results. Additionally, PROMs can alert health-care professionals for early detection of complications if specific PROMs results are low [6]. The delivery of PROMs commonly used in total hip and knee arthroplasties via mobile devices have been validated [[12], [13], [14]]. As such, the use of phone apps for patient monitoring and distribution of educational material is not a new concept in the industry. ePROMs provides flexibility for patient to complete PROMs at any location and in their own time. Consequently, it can reduce the number of outpatient visits to the clinical rooms as well as the need for patients to return completed PROMs via mail [15]. In the present study, it was found that 63% of participants preferred the ePROMs in the app over paper forms. Touvier et al. [16] similarly found participants preferring ePROMs over paper-based, with 92.2% of participants preferring the web-based version. Additionally, they had found that when questionnaires were completed by hand, 1.5% of entries had data entry mistakes and 1.1% with missing values. In comparison, the web-based questionnaires had faced none of these issues. Whilst the percentage of patients from the current study who had preferred the ePROMs from the app over paper-based was less than that of Touvier et al., there are a couple of factors that could have influenced the results. Firstly, the participants of the Touvier et al.’s study were provided with both the paper and web-based questionnaire and thus were able to make a direct comparison between the 2 methods, whilst the patients from the current study did not experience completing PROMs using the paper-based form. Secondly, the participants in Touvier et al.’s study were required to enroll online to be part of the study. Whilst Touvier et al. aimed to recruit patients who were not specifically trained web users, as the participants had to register through a website, the enrolled cohort may have had a higher level of experience and comfort with using technology in comparison to the joint arthroplasty candidates from the current study.

Despite the improved ease of access of PROMs through ePROMs, sustained compliance continues to be a challenge. Lyman et al. [17] conducted a study whereby an app was used to collect PROMs and track daily step counts following THA or TKA. At 6 months, they had found that 50% of THA and 43% of TKA patients did not complete the 6-month follow-up. Whilst most patients did not provide a reason for the noncompliance technological challenges were the most common reasons, including issues involved in downloading and navigating the app. Similarly, Smith et al. [18] conducted a study where ePROMs were used to send notifications via email or text with links to forms that the patients were required to complete. Reminder notifications were sent until the requested forms were completed or until the end of the eligible period. The patients were also encouraged to complete their PROMs at the follow-up appointments with the fellowship-trained shoulder surgeon. Despite these efforts, the compliance after 1 year was 58% and 51% at 2 years postoperatively. The study found that gender and age correlated with compliance, with younger males found to be less compliant. In this current pilot study, whilst no statistical correlations could be made, the general trend was similar to Smith et al., where male patients were found to be less compliant, with a compliance of 34%. However, there were no differences seen in age between the cohorts of compliant and noncompliant patients (65.9 vs 67.2 years, respectively). Abdeen et al. [19] reported on a nonrandomized prospective cohort study in adult patients undergoing primary elective Total Joint Arthroplasty (total enrolled 274: 139 App users and 135 non App users) to determine whether the use of a mobile App with timed reminders starting 5 days preoperatively, to perform a chlorhexidine gluconate shower and oral hydration protocol improves protocol adherence. They found that App-users had increased adherence to the hydration protocol (odds ratio 3.17 [95% confidence interval 1.42, 7.09: *P* = .003]). App-use was associated with shorter length of stay (median interquartile range 2.0 days [1.0, 2.0 days]) for App-users vs 2.0 days ([1.0, 3.0] for non-App users, *P* = .031). In their study, the authors observed App-users were more likely to be younger, male, and Caucasian and highlighted the potential inequity of access to the requisite technology. Equity rather than equality is an unmet need which needs urgent attention in particular when such mobile Apps are introduced. Indeed, the need for these is possibly the most for the underserved population who may have social, technical, financial, or cultural barriers to adopt these technologies. Sniderman et al. [20] in a recently published review, summarized the advantages and limitations of using a mobile app in patients undergoing THA or TKA. Sniderman et al. noted that Mobile applications have been associated with increased compliance to rehabilitation plans, and considerable cost savings when used appropriately. However, Sniderman et al. state that ongoing challenges exists regarding mobile app standardization, validation, equity, and cost.

The authors recognize limitations in the current study. The study was designed as a pilot study and thus a small study cohort was used. Consequently, statistical significance could not be made from the results of this study. As patients were reminded via a phone call when PROMs were missed, the reliability of the app alone in improving PROMs compliance could not be determined as such. Whilst phone call reminders can be implemented, this brings administrative burden, which can negate the advantages of using ePROMs. The study presents information based on a small patient cohort from a single center and those treated in the private set up. It is therefore not possible to extrapolate these results to other health care systems. As a result of screening patients for their ability to navigate a mobile phone, bias was introduced by filtering patients who were not diverse in socioeconomic backgrounds or literacy levels. In addition, patients recruited did not represent ethnic diversity and indeed acceptance of such an app in other ethnicities needs to be assessed. Cellular service can influence the outcome of patients living in rural areas. This study did not collect information regarding patient’s geographic location. As such, we cannot confirm whether cellular services had influenced the outcomes of patients in areas where there was low cellular coverage.

The 2 patients who withdrew their consent gave the reason that they were dissatisfied by the app. Since the primary objective of the study is satisfaction, bias is consequently introduced as these patients’ feelings of dissatisfaction are not represented in the mean satisfaction scores.

## Conclusions

A mobile phone application can be useful for both patient education and research data collection in appropriately selected patients. Whilst there are some patients for whom mobile phone technology is too difficult to use and paper forms will be still required, most patients (63%) reported electronic delivery of educational material and research questionnaires were preferable to a paper format. PROMs collected using a mobile phone app still caused some administrative burden as many participants required multiple reminders to complete their questionnaires.

## CRediT authorship contribution statement

**Leina Suzuki:** Writing – original draft, Visualization, Data curation. **Francis Connon:** Supervision, Methodology, Investigation, Funding acquisition, Conceptualization, Writing – review & editing. **Selin Munir:** Writing – original draft, Data curation. **Sarah Piplica:** Writing – review & editing, Project administration, Methodology, Investigation, Formal analysis, Data curation. **Hemant Pandit:** Writing – review & editing. **Daevyd Rodda:** Supervision, Methodology, Investigation, Funding acquisition, Conceptualization, Writing – original draft.

## Appendix A. Supplementary data

Conflict of Interest Statement for Rodda

Conflict of Interest Statement for Connon

Conflict of Interest Statement for Pandit

Conflict of Interest Statement for Suzuki

Conflict of Interest Statement for Munir

Conflict of Interest Statement for Piplica
