# Supplementary material for: Evaluation of a Mobile App to Assist Patient Education and Research in Arthroplasty
Source: Arthroplast Today. 2024 Nov 16;30:101549. doi: 10.1016/j.artd.2024.101549 (PMC11612646; doi:10.1016/j.artd.2024.101549)
Supplement: Appendix 1 [file mmc7.pdf]

QUESTIONNAIRE – Patient satisfaction survey

1. Overall, on a scale of 1 (not at all happy) to 5 (very happy), how happy were you with using the Patient Optimized Pathway Application (POP-App)?

Not at all happy	Not happy	Neutral happy	Happy	Very happy
1	2	3	4	5

2. Regarding your understanding of your rehabilitation requirements:
On a scale of 1 (not at all helpful) to 5 (very helpful), how would you rate the POP-App?

Not at all helpful	Not helpful	Somewhat helpful	Helpful	Very helpful
1	2	3	4	5

3. Regarding access to FAQs and educational materials:
On a scale of 1 (not at all helpful) to 5 (very helpful), how would you rate the POP-App?

Not at all helpful	Not helpful	Somewhat helpful	Helpful	Very helpful
1	2	3	4	5

4. Regarding filling out your patient surveys:
On a scale of 1 (not at all helpful) to 5 (very helpful), how would you rate the POP-App?

Not at all helpful	Not helpful	Somewhat helpful	Helpful	Very helpful
1	2	3	4	5

5. Regarding accessing your activity levels:
On a scale of 1 (not at all helpful) to 5 (very helpful), how would you rate the POP-App?

Not at all helpful	Not helpful	Somewhat helpful	Helpful	Very helpful
1	2	3	4	5

6. Would you have preferred to have your information and surveys delivered as paper handouts, on the App, or no difference?

Paper Handouts   No difference   POP-App

7. If you were to have this surgery again, would you use the App again?

No   Not sure   Yes
